# 59 Predictors and Impact of Pneumonia on Adverse Outcomes in Inhalation Injury Patients

**DOI:** 10.1093/jbcr/irad045.033

**Published:** 2023-05-15

**Authors:** Colette Galet, Karen Richey, Kevin Foster, Lucy Wibbenmeyer, Nicolas Ronkar

**Affiliations:** University of Iowa, Iowa City, Iowa; Arizona Burn Center Valleywise Health, Phoenix, Arizona; AZ Burn Center at Valleywise Health, Cave Creek, Arizona; University of Iowa, Iowa City, Iowa; University of Iowa, Iowa City, Iowa; University of Iowa, Iowa City, Iowa; Arizona Burn Center Valleywise Health, Phoenix, Arizona; AZ Burn Center at Valleywise Health, Cave Creek, Arizona; University of Iowa, Iowa City, Iowa; University of Iowa, Iowa City, Iowa; University of Iowa, Iowa City, Iowa; Arizona Burn Center Valleywise Health, Phoenix, Arizona; AZ Burn Center at Valleywise Health, Cave Creek, Arizona; University of Iowa, Iowa City, Iowa; University of Iowa, Iowa City, Iowa; University of Iowa, Iowa City, Iowa; Arizona Burn Center Valleywise Health, Phoenix, Arizona; AZ Burn Center at Valleywise Health, Cave Creek, Arizona; University of Iowa, Iowa City, Iowa; University of Iowa, Iowa City, Iowa; University of Iowa, Iowa City, Iowa; Arizona Burn Center Valleywise Health, Phoenix, Arizona; AZ Burn Center at Valleywise Health, Cave Creek, Arizona; University of Iowa, Iowa City, Iowa; University of Iowa, Iowa City, Iowa

## Abstract

**Introduction:**

Next to age and burn size, the presence of inhalation injury (II) is the third mortality prognostic factor for burn injury. II can lead to pulmonary complications such as pneumonia, acute respiratory distress syndrome (ARDS), and acute lung injury (ALI), all of which have been hypothesized to increase mortality in II. Herein, we aimed to identify variables associated with the risk of developing pneumonia and to determine the impact of pneumonia on selected outcomes.

**Methods:**

De-identified data from the Prospective Inhalation study (ISIS) were used. II was confirmed by fiberoptic bronchoscopy. Baseline demographics, injury data, burn size, laboratory data, radiographic results, treatment, and hospital course were recorded. Ventilator use, complications, including pneumonia, ARDS, ALI, and death, were tracked daily for seven days and then weekly until discharge. Univariate and multivariate analysis were performed. Variables included in multivariate analysis had a p value of 0.1 or less. A p value of < 0.05 was considered significant.

**Results:**

Of the 108 ISIS study group, 106 had complete data and were included in the analysis. Median age was 48.5 y, 59.4% of the subjects were male, 76.4% White and 9.4% Black. Regarding complications, 46.2% of the subjects developed pneumonia, 11.3% ARDS, 9.4% ALI, and 17% died. On univariate analysis, subjects who developed pneumonia were more likely to be white (87.8% vs. 66.7%, p = 0.006), to present with higher total burn surface area (24.70 [8.00-49.50] vs. 2.85 [0.00-25.38], p = 0.002), and full thickness burn (55.1% vs. 31.6%, p = 0.018). On multivariate analysis, being white (p = 0.033) and receiving colloids in the first 24 h of admission (OR = 3.582 [1.157-11.087], p = 0.027) were associated with increased risk of pneumonia while higher level of MMP-9 in the plasma (OR = 0.999 [0.998-1], p = 0.041) lowered the risk. Admission radiographs, FB score, clinical examination findings, and other demographic factors were not related to developing pneumonia. Pneumonia was independently associated with increased risk of developing ARDS (OR = 8.496 [1.322-54.61], p = 0.024) but not with ALI or mortality. Pneumonia was also associated with a longer hospital length of stay (B = 15.2 [2.4-28], p = 0.020) and higher number of days on a ventilator (B = 15.6 [8.2-23], p < 0.001).

**Conclusions:**

Pneumonia is associated with increased LOS and ventilator days but not mortality. Those who received colloids were 3.5 times more likely to develop pneumonia. The relationship between colloid and pneumonia requires more research, however, the search for other intervenable clinical variables to prevent pneumonia remains elusive.

**Applicability of Research to Practice:**

As colloids may affect the glycocalyx and contribute to inflammation, their role in developing pneumonia needs to be elucidated.

